# Supplementary material for: Identification of photoperiod-induced specific miRNAs in the adrenal glands of Sunite sheep (Ovis aries)
Source: Front Vet Sci. 2022 Jul 22;9:888207. doi: 10.3389/fvets.2022.888207 (PMC9354845; doi:10.3389/fvets.2022.888207)
Supplement: Supplementary file 1 [file Data_Sheet_1.ZIP › Supplementary file/miRDeep2 file.zip!/miRDeep2 file/Novel_Structure/LP42A1/Novel_346.pdf]

Provisional ID	:	NC_019469.2_21043
Score total	:	2.1
Score for star read(s)	:	-1.3
Score for read counts	:	0
Score for mfe	:	1.8
Score for randfold	:	1.6
Score for cons. seed	:	
Total read count	:	20
Mature read count	:	20
Loop read count	:	0
Star read count	:	0

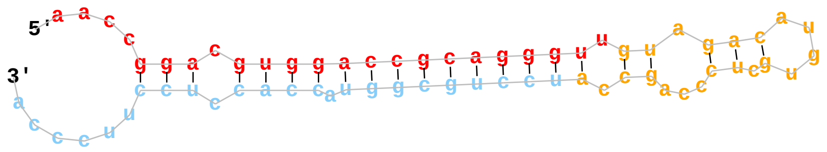

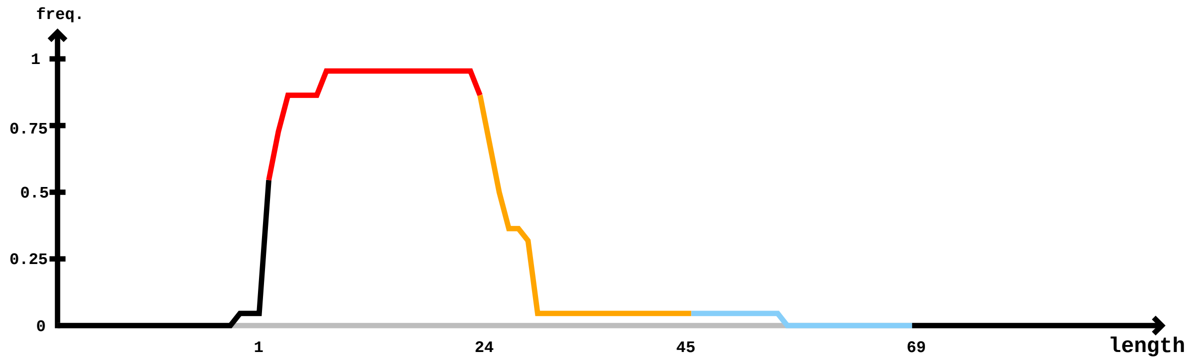

Mature Star

```
5'- acgacagcaggccaguucuaaaccggacguggaccgcaggguuguagacaugugcucccagccauccugcgguaccaccuccuucccagggagcccagugcagcucccagcgu -3'   exp
    ........................(((.((((((((((((((.((.(((....).))...)).)))))))))).)))).)))......((((((........)))))).....   reads   mm   sample
    .................cuaaaccggacguggaccgcaggguuguaga.................................................................   1       0    seq
    ....................aaccggacguggaccgcagggu.......................................................................   2       0    seq
    ....................aaccggacguggaccgcaggguu......................................................................   4       0    seq
    ....................aaccggacguggaccgcaggguug.....................................................................   2       0    seq
    ....................aaccggacguggaccgcaggguuguag..................................................................   1       0    seq
    ....................aaccggacguggaccgcaggguuguaga.................................................................   2       0    seq
    .....................accggacguggaccgcaggguu......................................................................   1       0    seq
    .....................accggacguggaccgcaggguugu....................................................................   2       0    seq
    .....................accggacguggaccgcaggguuguaga.................................................................   1       0    seq
    ......................ccggacguggaccgcaggguug.....................................................................   1       0    seq
    ......................ccggacguggaccgcaggguugu....................................................................   1       0    seq
    ......................ccggacguggaccgcaggguuguaga.................................................................   1       0    seq
    ..........................acguggaccgcaggguuguaga.................................................................   2       0    seq
    ................................................caugugcucccagccauccugcggua.......................................   1       0    seq
```
